# Whole-body total lesion glycolysis measured on fluorodeoxyglucose positron emission tomography/computed tomography as a prognostic variable in metastatic breast cancer

**DOI:** 10.1186/1471-2407-14-525

**Published:** 2014-07-21

**Authors:** Yoko Satoh, Atsushi Nambu, Tomoaki Ichikawa, Hiroshi Onishi

**Affiliations:** 1 PET Center, Kofu Neurosurgical Hospital, Sakaori 1-16-18, Kofu City, Yamanashi Prefecture, Japan; 2 Department of Radiology, Teikyo University School of medicine University Hospital, Mizonokuchi, Mizonokuchi 3-8-3, Takatsu, Kawasaki City, Kanagawa prefecture, Japan; 3 Department of Radiology, University of Yamanashi, Yamanashi University Faculty of Medicine, Shimokato 1110, Chuo City, Yamanashi prefecture, Japan

## Abstract

**Background:**

In this retrospective study, the authors evaluated the prognostic value of whole-body total lesion glycolysis (WTLG) on FDG/PET images in metastatic breast cancer (MBC) patients.

**Methods:**

We retrospectively evaluated 54 MBC patients who were diagnosed as having one or more metastatic lesions between June 2005 and March 2013. Twenty-four patients were diagnosed at the initial presentation (group A) and 30 patients were diagnosed for the first time at some point after a surgery (group B). Patients were excluded if they had received chemotherapy within 30 days before PET/CT. SUV_max_ and total TLG were calculated for all lesions in each patient and the highest SUV_max_ and the whole-body TLG (WTLG) values were used as independent variables for the analyses. Mean ages and the proportions of histopathological subtypes were compared between two groups using Mann–Whitney U test and Fisher’s exact test, respectively. The prognostic significance of PET parameters was assessed using Cox proportional hazards regression analysis.

**Results:**

For groups A and B, the median follow-up period was 26 months (range, 3–58 months) and 40.5 months (range, 3–69 months), and the median age was 61 years (range, 42–81 years) and 59 years (range, 24–74 years), respectively. There were no significant differences between two groups in age (p = 0.294) or histopathological subtype (p = 0.384). In the univariate analyses, WTLG was found to be significantly associated with overall survival (OS) for patients of group A (p = 0.012). In the multivariate analysis, WTLG was also significantly associated with OS (p = 0.015). Only hormonal receptor level was a significant indicator of longer OS in patients with recurrent MBC (group B).

**Conclusions:**

This study demonstrated that WTLG on PET/CT is an independent prognostic factor for survival in breast cancer patients with metastases at the initial presentation.

## Background

Breast cancer is now the most common cancer and is one of the leading causes of cancer-related death among women [1]. Breast cancer mortality has been declining due to earlier detection of the disease and treatment that is more effective than before [2]. However, despite improvements in the treatment of breast cancer over the last decade, metastatic breast cancer (MBC) remains a significant public health problem, since of the 1 million women worldwide who are diagnosed annually with early-stage breast cancer, approximately 33% will eventually experience relapse [3]. For these MBC patients, prognostic information is crucial in order to facilitate appropriate therapies for improving quality of life and prolonging survival. Recently, not only hormone receptors, such as estrogen receptor or progesterone receptor (ER/PR), but also biomarkers that regulate tumor cell proliferation, such as the Ki67 index or human epidermal growth factor receptor type 2 (HER2) status, have been introduced as predictive indicators for prognosis [4]. Since these indicators are derived from tissue specimens from primary breast tumors at the time of first diagnosis, they do not represent the whole extent of disease. Therefore, more reliable prognostic indicators that can more accurately reflect the extent as well as malignancy of the disease in MBC patients are required.

Positron emission tomography (PET) has emerged as an important molecular imaging technique in the evaluation and clinical management of a number of neoplasms. Fluorine-18-fluorodeoxyglucose (FDG) PET has already achieved wide acceptance for use in the initial staging and restaging of cancers, the evaluation of recurrence, and the monitoring of treatment effects in patients with cancers, because its sensitivity and specificity for diagnosing malignancy are better than those of other imaging methods. Furthermore, the standardized uptake value (SUV) of primary tumors has already been shown to be of value as a prognostic indicator of survival in patients with operable breast cancer [5]. FDG PET may also allow measurement of the tumor burden in relation to the degree of malignancy. The high tumor-to-background intensity ratios in FDG PET enable computer-assisted measurement of total body metabolic tumor volume or total lesion glycolysis (TLG). As a prognostic factor in MBC patients, FDG PET evaluations may be more advantageous over assessments involving hormone receptors or biomarkers that regulate tumor cell proliferation, which require tissue sampling. This is because FDG PET can directly evaluate the extent of metastatic disease as well as total tumor burden without biopsy. In addition, as volume is not one of the constituents of SUV, TLG may be a better prognostic factor than SUV. Thus, the PET parameters of metastatic sites in MBC patients at the initial presentation, or at the time of recurrence, may provide important information about the aggressiveness of whole tumors that could be of prognostic significance. Therefore, the objective of this study was to evaluate the predictive performance of TLG-based FDG PET/CT assessments in MBC patients.

## Methods

This study was approved by the Ethics Committee of the Kofu Neurosurgical Hospital. All patients gave written informed consent for future anonymous use of clinical data in clinical study.

### Patients

We conducted this retrospective study of patients with breast cancer who underwent FDG PET/CT between June 2005 and December 2012 at a single institution. A total of 148 breast cancer patients were suspected of having metastatic disease on the basis of FDG PET/CT assessments. Medical records were reviewed to gather clinical data, including information on age, histology, tumor phenotype (ER, PR, and HER2 expression), and final outcome.

**Figure 1:**
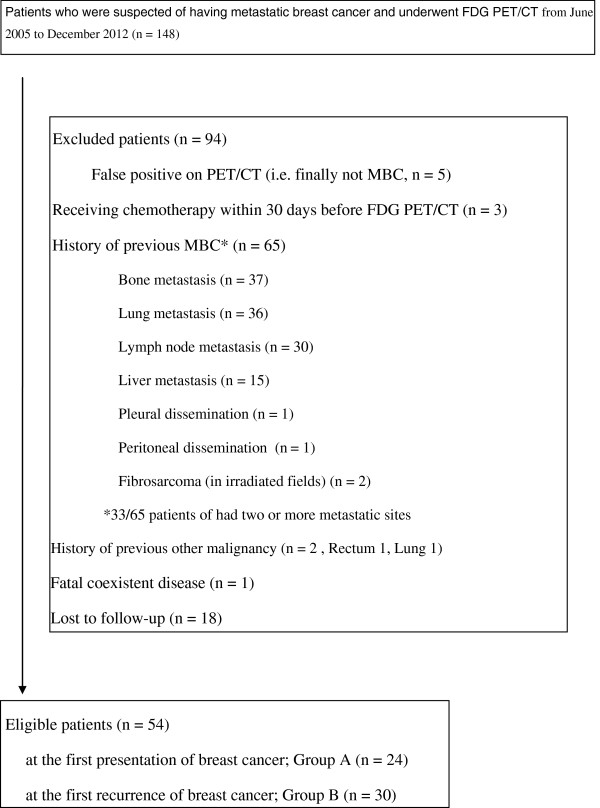
**Reasons for exclusion of patients and the number of eligible patients.** The reasons for exclusion and the number of eligible patients are shown in the figure. Abbreviations: FDG, [^18^ F]-2-fluoro-2-deoxy-D-glucose; PET/CT, positron emission tomography/computed tomography; MBC, metastatic breast cancer.

PET/CT images were obtained within 60 days of MBC diagnosis in all patients. The date of MBC diagnosis was defined as the date when the patient underwent biopsy of a metastatic site or the date of radiological examinations at the first confirmed metastasis. Patients who already had shown MBC, a history of other malignancies, or had received chemotherapy within 30 days before PET/CT imaging were excluded. However, receiving endocrine therapies during this period was not considered a criterion of exclusion. The reasons for exclusion of patients are summarized in Figure 1. A total of 54 MBC patients were eventually eligible for this study. Twenty-four patients were at the initial presentation stage (group A) and the remaining 30 patients were at the first recurrence stage (i.e., newly diagnosed as having MBC after a surgery; group B). Table 1 summarizes patient characteristics, tumor phenotypes, and initial clinical stages.

**Table 1:** Clinical characteristics of the study subjects

	Group A* (n = 24)	Group B** (n = 30)	**p value**
**Age (median, range)**	**61, 42-81**	**59, 24-74**	**0.294**
Tumor phenotype			
ER/PR positive and HER2 negative	14	19	0.783
HER2 positive	5	5	0.695
Triple negative	2	6	0.277
Unknown	3	0	0.081
Histology			0.384
Ductal	19	26	
Lobular	2	0	
Others	0	1	
Unknown	3	3	
Initial clinical stage			
I	-	6	
II	7	15	
III	8	6	
IV	8	2	
unknown	1	1	
Follow-up period (months)			
Median (range)	26 (3–58)	40.5 (9–69)	

*Group A: Cases found to have metastases at the initial presentation.

**Group B: Cases found to have newly diagnosed metastases at some point after a surgery.

*Abbreviations:ER* estrogen receptor, *PR* progesterone receptor, *HER* human epidermal growth factor 2.

The diagnosis of MBC was based on histopathological diagnosis of a metastatic site or the findings of conventional imaging examinations, such as ultrasound, bone scan, CT, or magnetic resonance imaging. As for the imaging diagnoses of MBC, the diagnostic criteria were based on the presence of the following lesions: newly appearing lesions on a follow-up examination; lesions increasing in size; or lesions that had decreased in size on the follow-up examinations after additional treatments, such as chemotherapy, radiation therapy, or molecular targeted drug therapy. The median follow-up periods were 26 months (range, 3–58 months) and 40.5 months (range, 9–69 months), and the median ages were 61 years (range, 42–81 years) and 59 years (range, 24–74 years), for group A and B after the diagnoses of MBC, respectively.

### PET/CT

All patients fasted for at least 6 hours before the FDG-PET examination, although oral hydration with glucose-free water was allowed. After peripheral blood glucose level was confirmed to be <150 mg/dL, patients received an intravenous injection of 3 MBq/kg of FDG. The FDG used for the PET scans was produced at the biochemical cyclotron facility in the PET center at Kofu Neurosurgical Hospital. All whole-body PET/CT images were obtained using a PET/CT scanner (Biograph Duo LSO; Siemens Medical Solutions; Erlangen, Germany) consisting of a PET scanner and a 2-detector row CT scanner. The axes of both systems were mechanically aligned so that the patient could be moved from the CT scanner to the PET scanner gantry. Thus, the resulting PET and CT scans co-registered on the same hardware. PET/CT scanning was performed from the center of the skull to the upper thigh 60 min after the injection. CT was performed using the following settings: 110 kVp; 30 mA; tube rotation time, 0.8 s per rotation; beam pitch, 2; transverse field of view, 50 cm; and section thickness, 2.5 mm. The patients maintained normal shallow respirations during the CT scans. No iodinated contrast material was administered. The PET scans were performed immediately after the CT scans, with an identical transverse field of view. The PET scans were acquired in a 3-dimensional mode with a matrix size of 128 × 128. After the transmission scan, the PET acquisition time was 2 min for each table position. The CT data was resized, from a 512 × 512 matrix to a 128 × 128 matrix, to match the PET data, so that the scans could be fused and CT-based transmission maps could be generated. PET data sets were iteratively reconstructed using an ordered subset expectation maximization algorithm and segmented attenuation correction (2 iterations, 8 subsets). Integrated, co-registered PET/CT images on three orthogonal (transaxial, coronal and sagittal) planes were reviewed on a workstation (e-soft-PET; Siemens Medical Solutions), which enabled image fusion and analysis.

### Measurement of PET parameters

Experienced nuclear medicine physicians prospectively interpreted PET/CT images with other available imaging examinations (e.g., previous CT). FDG uptake was evaluated on the basis of a region-of-interest analysis.

A nuclear medicine physician (Y.S.) with 10 years’ experience in PET/CT interpretation analyzed all FDG PET/CT images semi-quantitatively by using dedicated software (e-soft-PET; Siemens Medical Solutions).

All lesions were evaluated separately. The SUVs were acquired using attenuation-corrected images. From PET/CT scans, the maximum SUV (SUVmax) and metabolic tumor volume (MTV) were calculated. The software created a 3D contour around voxels that were equal to or greater than 50% of the maximum voxel value inside the spherical region. This volume was defined as the MTV. The threshold of 50% was used in accordance with the findings of a prior publication [6]. Among the SUVmax values of all the lesions in each patient, the highest SUVmax (HSUVmax) value was used for the analysis. Total lesion glycolysis (TLG) was calculated as MTV multiplied by the mean SUV (TLG = MTV × mean SUV). Whole-body TLG (WTLG) was calculated as the sum of the TLG values of all lesions in each patient.

### Statistical analysis

Statistical analysis was performed using the PASW Statistics 18 (SPSS; Chicago, USA).

Mean ages and the proportions of histopathological subtypes and phenotypes were compared between the two groups using Mann–Whitney U test and Fisher’s exact test, respectively. No correction for multiple comparisons was made. Univariate and multivariate analyses were performed to evaluate the effects of the highest SUVmax, WTLG, and other clinical variables on overall survival (OS), using Cox proportional hazards regression analysis. Overall survival was defined as the period of time from the day of diagnosis of MBC to the day of death or final clinical follow-up. In group A, where the primary breast cancers of these patients had not been resected, additional analyses were performed including SUVmax of the primary breast cancers as an independent variable.

Since independent variables with a high correlation coefficient may lead to multicollinearity, correlations between the independent variables in the multivariate analyses had been calculated in advance, using the Pearson’s correlation coefficient or Spearman’s rank correlation coefficient. Survival rates were calculated using the Kaplan–Meier method. All tests were 2-sided, and p values less than 0.05 were considered statistically significant.

## Results

There were no significant intergroup differences in age (p = 0.393), histopathological subtype (p = 0.384), and phenotypes: ER/PR-positive and HER2-negative (p = 0.783), HER2-positive (p = 0.695), and triple-negative (p = 0.277).

Median HSUVmax and WTLG were 6.32 (range, 1.72-15.21) and 59.92 (range, 3.62-1904.3) for group A, and 4.48 (range, 1.28-12.06) and 12.38 (range, 0.7-243.64) for group B, respectively. The numbers of patients with metastases in anatomic sites are shown in Table 2. The mean number (range) of metastases was 2.3 (1–10) and 2.2 (1–7) in groups A and B, respectively. In group A, no metastatic lesion was diagnosed histopathologically prior to treatment. Although all patients treated with surgery (16/24) in group A underwent histopathological assessments to confirm if they had axillary lymph node metastasis, pretreatment imaging findings were used for the analyses in patients who received neoadjuvant chemotherapy (9/16). In group B, 6 metastatic lesions (axillary lymph node: 4, subclaviclar lymph node: 1, supraclaviclar lymph node: 1) were histopathologically diagnosed.

**Table 2:** Numbers of patients showing metastasis at various sites

**Site of metastasis**	Group A* (n = 24)	Group B** (n = 30)
Axillary lymph node	22 (91.7%)	7 (23.3%)
Extra-axillary lymph node	7 (29.2%)	13 (43.3%)
Bone	4 (16.7%)	9 (30%)
Liver	1 (4.2%)	2 (6.7%)
Lung	3 (12.5%)	4 (13.3%)
Pleural dissemination	0 (0%)	1 (3.3%)

*Group A: Cases found to have metastases at the initial presentation.

**Group B: Cases found to have newly diagnosed metastases at some point after a surgery.

In the univariate and multivariate analyses for group A, WTLG was a significant predictor of OS, while HSUVmax and tumor phenotype (ER/PR-positive and HER2-negative, and triple negative) were not statistically significant predictors of OS (Table 3). On the other hand, in the univariate and multivariate analyses for group B, the ER/PR-positive and HER2-negative phenotype were significant predictors while HSUVmax and WTLG were not significant predictors (Table 3).

**Table 3:** Univariate and multivariate analyses for overall survival in group A* using Cox model

	**HR**	**95% CI**	**p**
Univariate analysis			
Age	0.989	0.927-1.054	0.727
ER/PR(+)	0.203	0.033-1.248	0.085
highest SUVmax	1.145	0.895-1.466	0.282
WTLG	1.004	1.001-1.006	0.012*
Multivariate analysis			
ER/PR(+)	0.273	0.017-4.329	0.357
highest SUVmax	0.792	0.503-1.245	0.312
WTLG	1.004	1.0011.007	0.015*

*Group A: Cases found to have metastases at the initial presentation.

*Abbreviations:ER* estrogen receptor, *PR* progesterone receptor, *SUV* standardized uptake value, *WTLG* whole-body total lesion glycolysis.

SUVmax of primary breast cancer was not a significant predictor for OS in the univariate and multivariate analyses for group A (p = 0.136, 0.429, Additional file 1: Table S4). Tumor staging was not a significant predictor for OS in both univariate and multivariate analyses (data not shown). Correlation coefficients between age, tumor phenotype (ER/PR-positive and HER2-negative), HSUVmax, and WTLG are shown in Additional file 2: Table S5. At 3 years, OS of group A and group B was 77.4% and 86.4%, respectively.

## Discussion

Prognostic factors used in breast cancer can broadly be divided into those that determine the extent of disease (i.e., tumor staging) and those that determine biological tumor characteristics. The latter are now crucial not only in predicting prognosis but also in selecting an appropriate treatment regimen [7-9]. In addition, the usefulness of PET parameters in breast cancer has also been reported currently [5,10,11]. However, although many authors have advocated that the SUVmax of FDG PET is an important predictor of progression after treatment, the clinical importance of SUVmax as a prognostic factor for malignancy is still controversial [12,13]. Because SUVmax is a single-voxel-based value, it may not be an adequate surrogate marker for the true biology of the whole tumor. In addition, SUVmax is susceptible to statistical noise and thus may be an unstable parameter. On the other hand, it has been reported that volume-based parameters (e.g., metabolic tumor volume, MTV, TLG, etc.) were significantly associated with an increased risk of recurrence and death in patients with surgically resected non-small cell lung cancer [14].

Volume-based parameters of FDG PET represent the metabolic tumor burden of disease, reflecting both tumor volume and glucose utilization rate. These indices have been considered to be potentially reliable parameters for providing more details about the status of diseases in various types of cancers, especially lung tumors, oro/nasopharyngeal, and rectal cancers [15-18]. Most recently, some authors have reported that TLG was superior to SUVmax as a predictor of prognosis in malignancies [19,20]. Our results showed that the WTLG of MBC in the initial presentation (group A) was correlated with OS not only in the univariate analysis but also in the multivariate analysis after adjustments for tumor phenotype and HSUVmax. Since ER/PR status was not a significant prognostic factor in our analyses, it could be said that WTLG is a better predictor of prognosis than ER/PR status in MBC patients at the initial presentation. Furthermore, SUVmax of primary breast cancers was not a significant predictor of OS in the multiple variable analysis as well as in the univariate analysis. Thus, WTLG may be considered a better prognostic factor than SUVmax in primary breast cancer patients who show metastatic disease at the initial presentation.

Conversely, the univariate and multivariate analyses for group B identified only ER/PR status as an independent prognostic factor. This result might suggest that WTLG does not accurately represent the extent of disease in recurrent breast cancers. This result may be explained by considering the finding that a similar number of patients in group B whose tumors were ER/PR positive had received endocrine therapy (14/20 [70%], aromatase inhibitor or antiestrogen). At the time of FDG PET/CT, FDG accumulation in MBC may have been diminished by the effects of these hormonal drugs, when compared with FDG accumulation in MBC at the first presentation. FDG PET/CT may be inappropriate for estimating the inherent malignancy or the extent of MBC when patients are receiving chemotherapy, including endocrine therapy. Morris et al. [21] reported that the SUVmax of the newly diagnosed MBC to bone was significantly correlated with OS, and with the same group of patients, Ulaner GA et al. [22] also have recently reported that the TLG tertile in the multivariate analysis was significantly associated with OS in patients with bone metastasis. We did not perform individual analyses for each metastatic site because of the insufficient sample size in our study. While their observations were inconsistent with our results that TLG was not a significant prognostic factor in patients with recurrence after a surgery (group B), this difference might be caused by the smaller sample size.

There were a few limitations to our study. First, because of the retrospective nature of this study, the patient population in our study was heterogeneous in terms of follow-up strategy (e.g., follow-up period and choice of imaging modality) and treatment regimens. Second, the number of the study subjects was relatively small, as we mentioned before.

## Conclusions

In summary, the present study suggested that a higher WTLG, as a volumetric parameter of FDG PET, predicts worse OS in MBC patients at their initial presentation. A further prospective study to validate our results would be warranted.

## Competing interests

The authors declare that they have no competing interests.

## Authors’ contributions

YS collected and reviewed the clinical information, carried out the PET analysis, and conducted the statistical analysis. YS and AN conceived the study and drafted the manuscript. TI and HO developed the study design. All authors have read and approved the final manuscript.

## Pre-publication history

The pre-publication history for this paper can be accessed here:

http://www.biomedcentral.com/1471-2407/14/525/prepub

## Supplementary Material

Additional file 1 Univariate and multivariate analyses for overall survival in group A* using Cox model (with SUVmax of primary tumor as an independent variable). Click here for file

Additional file 2 Correlation coefficients between each dependent variable. Click here for file
